# A Mathematical Model for the Hydrogenotrophic Metabolism of Sulphate-Reducing Bacteria

**DOI:** 10.3389/fmicb.2019.01652

**Published:** 2019-07-17

**Authors:** Nick W. Smith, Paul R. Shorten, Eric Altermann, Nicole C. Roy, Warren C. McNabb

**Affiliations:** ^1^AgResearch, Ruakura Research Centre, Hamilton, New Zealand; ^2^AgResearch, Grasslands Research Centre, Palmerston North, New Zealand; ^3^Riddet Institute, Massey University, Palmerston North, New Zealand; ^4^School of Food and Advanced Technology, Massey University, Palmerston North, New Zealand; ^5^High-Value Nutrition National Science Challenge, The University of Auckland, Auckland, New Zealand

**Keywords:** hydrogen, gastrointestinal tract, IBS, hydrogen sulphide, mathematical modelling

## Abstract

Sulphate-reducing bacteria (SRB) are studied across a range of scientific fields due to their characteristic ability to metabolise sulphate and produce hydrogen sulphide, which can lead to significant consequences for human activities. Importantly, they are members of the human gastrointestinal microbial population, contributing to the metabolism of dietary and host secreted molecules found in this environment. The role of the microbiota in host digestion is well studied, but the full role of SRB in this process has not been established. Moreover, from a human health perspective, SRB have been implicated in a number of functional gastrointestinal disorders such as Irritable Bowel Syndrome and the development of colorectal cancer. To assist with the study of SRB, we present a mathematical model for the growth and metabolism of the well-studied SRB, *Desulfovibrio vulgaris* in a closed system. Previous attempts to model SRB have resulted in complex or highly specific models that are not easily adapted to the study of SRB in different environments, such as the gastrointestinal tract. We propose a simpler, Monod-based model that allows for easy alteration of both key parameter values and the governing equations to enable model adaptation. To prevent any incorrect assumptions about the nature of SRB metabolic pathways, we structure the model to consider only the concentrations of initial and final metabolites in a pathway, which circumvents the current uncertainty around hydrogen cycling by SRB. We parameterise our model using experiments with varied initial substrate conditions, obtaining parameter values that compare well with experimental estimates in the literature. We then validate our model against four independent experiments involving *D. vulgaris* with further variations to substrate availability. Further use of the model will be possible in a number of settings, notably as part of larger models studying the metabolic interactions between SRB and other hydrogenotrophic microbes in the human gastrointestinal tract and how this relates to functional disorders.

## Introduction

Sulphate-reducing bacteria (SRB) play an important role in a variety of ecosystems, from marine sediments and oil fields to the human gastrointestinal tract (Muyzer and Stams, 2008; Carbonero et al., 2012a). The functional group of SRB has been reported to comprise 60 genera (Barton and Fauque, 2009), and is characterised by the ability to utilise sulphate as an electron acceptor during metabolism. The presence of these bacteria has both positive and negative implications on human activities, depending on the context. Much research has been performed on hydrogen sulphide (H_2_S) production in oil fields by SRB, which can lead to reduced oil quality and machinery corrosion (Magot et al., 2000), and in the treatment of industrial wastewater, as the sulphides SRB produce facilitate the removal of contaminating heavy metals (Kiran et al., 2017). Less clear are the implications of SRB in the human gastrointestinal tract (GIT). The SRB population size in the GIT has been measured at approximately 10^7^ cells per gram of faeces (Doré et al., 1995), but varies between individuals (Nava et al., 2012) and between studies (Smith et al., 2018). These bacteria are widely studied due to their controversial role in a number of functional GIT disorders. Increased levels of colonic SRB and increased H_2_S concentrations have been linked to Irritable Bowel Syndrome, Inflammatory Bowel Disease and colorectal cancer [for a review, see Carbonero et al. (2012b)]. However, beneficial effects of H_2_S have also been investigated, such as its capacity to stimulate mucus production (Motta et al., 2015) and the potential influence of this molecule on blood pressure regulation (Tomasova et al., 2016). The important connexions between SRB, H_2_S and the host justify further research into the metabolism of these bacteria.

Another key molecule in SRB metabolism is elemental hydrogen. Alongside methanogens and reductive acetogens, SRB can metabolise free hydrogen present in the GIT, utilising it in the reduction of sulphate (Smith et al., 2018). The sulphate metabolised by SRB can be dietary or host-derived; cross-feeding by SRB on sulphate released during mucin metabolism by other GIT microbes has been well studied (Willis et al., 1996; Rey et al., 2013). High concentrations of hydrogen in the GIT are known to inhibit the metabolism of saccharolytic members of the microbiota (Wolin and Miller, 1983), therefore the presence of hydrogen cross-feeders is thought to increase the rate of carbohydrate breakdown by the wider microbial population. This has been shown in rodent models and linked to increased energy yield for the host (Samuel and Gordon, 2006; Rey et al., 2010).

Due to the importance of SRB in human health and nutrition, a greater understanding of their metabolism and growth dynamics is sought. To this end, we developed a mathematical model for the metabolite flux and population growth of the human SRB *Desulfovibrio vulgaris*, grown on substrates found in the GIT (Scanlan et al., 2009). Ours is not the first attempt to model SRB metabolism and growth and we compare the predictions of our model with that of the existing mathematical model of Noguera et al. (1998). Many other mathematical models of SRB have been published, but these are almost universally applied to address specific characteristics of SRB or for the investigation of competitive and syntrophic relationships between SRB and methanogens [for example, Robinson and Tiedje (1984), Okabe et al. (1995), Stolyar et al. (2007)]. The model of Noguera et al. (1998) is not targeted to a specific characteristic or environment, therefore is a good benchmark against which to compare our model. The existing model is more complex than that proposed here: it consists of ten ordinary differential equations for aqueous and gaseous metabolite concentrations and microbial growth and is dependent on 20 parameter values that are estimated either from separate experimental work or from model fitting. While the model considers many aspects of the metabolism of *D. vulgaris*, it is computationally intensive and requires greater knowledge of kinetic parameters than is often available in environments such as the GIT. Therefore, its structure is less readily compared or combined with other existing models for the GIT microbiota. We also found that this model shows sensitivity to the initial values for dissolved hydrogen and carbonate concentrations; values that are difficult to determine experimentally and physiologically. As we wish to study SRB in the GIT, we construct a simpler model requiring less inputs to later integrate into a larger microbiota model. Our SRB model considers solely the concentrations of the initial and final metabolites in a metabolic pathway, treating the intermediate metabolites and reactions as a “black box.” We calibrate our model using existing experimental data for the monoculture growth of a *D. vulgaris* strain and use it to predict the dynamics of separate independent experiments with both the same bacterium and a different *D. vulgaris* strain.

## Materials and Methods

### Assumptions

For this model it was assumed that the only metabolites involved in the metabolism of *D. vulgaris* are lactate, acetate, hydrogen, sulphate and hydrogen sulphide (H_2_S), as these metabolites represent important initial and final metabolites in the major metabolic pathways of *D. vulgaris* (Keller and Wall, 2011). Other metabolic pathways involving fermentation of alternative organic molecules, such as monosaccharides and fatty acids, and reduction of nitrogenous compounds have been studied in *Desulfovibrio* and other SRB genera, but appear to be of lesser importance and not widespread within the functional group (Barton and Fauque, 2009). While formate has been implicated in the metabolism of *Desulfovibrio* species elsewhere (da Silva et al., 2013; Junicke et al., 2015; Martins et al., 2015), here we have assumed that formate may be represented as hydrogen equivalents. This is supported by the similar reduction potentials of formate and hydrogen, allowing for interconversion of the two molecules at low energetic cost to the bacterium (Stams and Plugge, 2009; da Silva et al., 2013; Rabus et al., 2013). Formate concentrations also remained very low (<0.5 mM) in previous experiments with *D. vulgaris* Hildenborough grown on either lactate and sulphate or lactate and hydrogen (da Silva et al., 2013).

We assume that the medium in which *D. vulgaris* is grown contains in abundance all other molecules necessary for growth and that these are not significantly depleted during the experiment. We further assume that *D. vulgaris* is able to oxidise lactate incompletely to acetate, with concurrent production of hydrogen (Keller and Wall, 2011). This hydrogen may then be utilised in the reduction of sulphate to H_2_S. We assume that all metabolites remain in the aqueous phase, with the exception of hydrogen, which may transfer between the aqueous and gaseous phases. We assume that all metabolites in the aqueous phase are available to the bacteria in a well-mixed solution. No spatial component is considered in the model.

The assumed stoichiometries for the two reactions, expressing all protons as hydrogen molecule equivalents, are as follows (Thauer et al., 1977; Noguera et al., 1998; da Silva et al., 2013):

CHCHOHCOO3(Lactate)-+2HO2 →CHCOO3(Acetate)-+2.5H+2HCO-3

SO(Sulphate)42-+5H→2HS2+4HO2

Note that the bicarbonate molecule (HCO_3_^−^) produced in the oxidation of lactate and the water molecules produced in the reduction of sulphate are not included in the model, as they play no further role in the metabolism of *D. vulgaris*. Moreover, we assume that the culture remains well buffered throughout the experiment, therefore pH is not altered by changing concentrations of bicarbonate or other metabolites. There have been reports of bicarbonate as a growth-limiting molecule for other bacterial strains (Dobay et al., 2018), but there is currently no evidence of this for SRB. We explain this further in the Discussion.

### Mathematical Model

**FIGURE 1:**
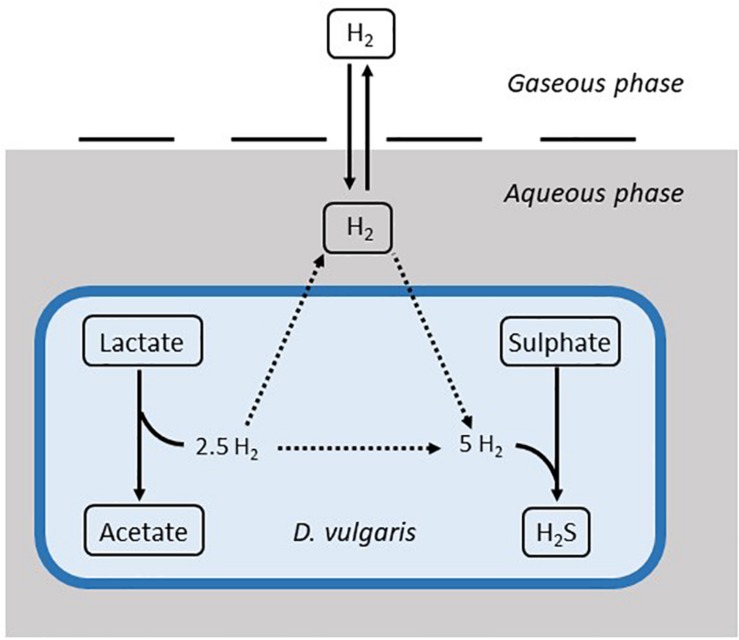
Structure of the mathematical model. Solid arrows denote modelled dynamics. Dotted arrows denote dynamics that are not explicitly modelled. H_2_S: hydrogen sulphide.

The model is based on Monod kinetics for bacterial growth in a batch culture environment (Monod, 1949). Monod kinetics was chosen due to the biological meaning associated with the parameters, as well as the ability to determine these values experimentally if required. The model considers the molar concentration of lactate, acetate, sulphate and H_2_S, as well as the molar concentration of hydrogen in the aqueous phase and the partial pressure of hydrogen in the gaseous phase, measured in atmospheres. It also considers the concentration of the bacterial population in the aqueous phase (mg L^–1^). These units were chosen to align with data sources for both the calibration and validation of the model. Figure 1 shows the general structure of the model.

Following Monod kinetics, we model the rate of change in lactate concentration (*L*; mM) by

(1) $$\frac{d\,L}{d\,t}=-\frac{\mu_{m\,a\,x,L}\,X}{Y_{L}}\,\left(\frac{L}{K_{L}+L}\right)$$

where μ_*max,L*_ denotes the maximum growth rate (h^–1^) and *Y*_*L*_ denotes the biomass yield (mg L^–1^ mM^–1^) of *D. vulgaris* when grown on lactate. *K*_*L*_ is the Monod constant (mM) for this bacterium and substrate, also referred to as the half-saturation constant. This value is the concentration of substrate required for the bacterium to attain half of its maximum growth rate. *X* is the concentration of bacterial cells in the medium (mg L^–1^).

It is known that high concentrations of hydrogen in the medium inhibit the metabolism of lactate by certain SRB, including *D. vulgaris*, although the mechanism is not clear (Pankhania et al., 1988; Junicke et al., 2015). As such, we add an inhibition term to our model that reduces the rate of lactate metabolism as the aqueous hydrogen concentration, *H*_*aq*_ (mM), increases. Equation 1 then becomes

(2) $$\frac{d\,L}{d\,t}=-\frac{\mu_{m\,a\,x,L}\,X}{Y_{L}}\,\left(\frac{L}{K_{L}+L}\right)\,\left(1-\frac{H_{a\,q}}{H_{m\,a\,x}}\right)$$

where *H*_*max*_ (mM) is the aqueous hydrogen concentration above which lactate degradation is completely inhibited. This formulation also ensures that the rate of lactate degradation reduces proportionally to the aqueous hydrogen concentration. To ensure that the model is robust to hydrogen concentrations above *H*_*max*_, we add the following condition:

$$\frac{d\,L}{d\,t}=0\;\text{when}\,H_{a\,q}>H_{m\,a\,x}.$$

The sulphate concentration (*S*; mM) is given by

(3) $$\frac{d\,S}{d\,t}=-\frac{\mu_{m\,a\,x,S}\,X}{Y_{S}}\,\left(\frac{S}{K_{S}+S}\right)\,\left(\frac{H_{a\,q}}{K_{H}+H_{a\,q}}\right).$$

Sulphate and hydrogen are both required for the formation of H_2_S, hence the inclusion of the aqueous hydrogen concentration in Eq. 3. The equation is adapted from the model equations of Kettle et al. (2015) for multiple essential resources. μ_*max,S*_ denotes the maximum growth rate (h^–1^) and *Y*_*S*_ is the biomass yield (mg L^–1^ mM^–1^) of *D. vulgaris* during sulphate reduction. *K*_*S*_ and *K*_*H*_ denote the Monod constants (mM) for sulphate and hydrogen, respectively.

We assume that the aqueous hydrogen concentration is influenced by hydrogen production during the oxidation of lactate, hydrogen consumption in the reduction of sulphate, and liquid-gas transfer of hydrogen. The rate of change in the concentration of aqueous hydrogen is

(4) $$\frac{d\,H_{a\,q}}{d\,t}=-b_{L\,H}\,\frac{d\,L}{d\,t}+b_{H\,P}\,\frac{d\,S}{d\,t}-\frac{1}{\rho_{H}}\,\frac{d\,H_{g}}{d\,t}\,\frac{V_{g}}{V_{a\,q}}$$

where *b*_*LH*_ is the stoichiometric constant for moles of hydrogen produced per mole lactate metabolised and *b*_*HP*_ is the stoichiometric constant for moles of hydrogen required to reduce one mole of sulphate. *H*_*g*_ is the gaseous hydrogen concentration, measured in atmospheres, and mass transfer between the aqueous and gaseous phases is assumed to be linear, with

(5) $$\frac{d\,H_{g}}{d\,t}=k_{L}\,a\,\left(\rho_{H}\,H_{a\,q}-H_{g}\right)\,\frac{V_{a\,q}}{V_{g}}$$

Equation 5 represents a simple mass transfer model as explained in Kadic and Heindel (2014). Briefly, net transfer between the two phases is determined by the concentration gradient, with the rate of transfer determined by the mass transfer coefficient, *k*_*L*_ (calculated from the thickness of the film through which molecules must travel and the diffusivity of the molecule in question) and the surface area, *a*, across which mass transfer may occur. Although other, more complex models do exist for mass transfer between two phases, as only the gaseous hydrogen concentration data is available here, we are limited in our ability to parameterise a more complex model. Although the simplicity of this representation may result in sub-optimal representation of the hydrogen dynamics, we also seek to minimise the number of fitted parameter values in our model, and thus the film model described here is sufficient for our purposes. *k*_*L*_*a* has the unit h^–1^ and *V*_*g*_ and *V*_*aq*_ (mL) are the fixed volumes of the gaseous and aqueous phases, respectively. ρ_*H*_ (atm mM^–1^) is the Henry conversion constant for hydrogen. *H*_*g*_ is measured in atmospheres, whereas *H*_*aq*_ is given in mM concentration, therefore we adapt the gas transfer equation used in Muñoz-Tamayo et al. (2016) for our model, giving a ρ_*H*_ value of 1.364 atm mM^–1^.

The rates of change in acetate (*A*) and H_2_S (*P*) concentrations are proportional to the rates of change in the concentrations of lactate and sulphate, respectively.

(6) $$\frac{d\,A}{d\,t}=-b_{L\,A}\,\frac{d\,L}{d\,t}$$

(7) $$\frac{d\,P}{d\,t}=-b_{S\,P}\,\frac{d\,S}{d\,t}$$

where *b*_*LA*_ and *b*_*SP*_ are constants determined by the stoichiometries of each reaction stated in Section 2.1. Note that we take these stoichiometries directly from the literature and do not include in the model some fraction of substrate being used in the production of cell biomass. This assumption is made as, for the batch culture cases considered here, the experimentally observed stoichiometries of the metabolites closely matched those given in Section 2.1.

Finally, the concentration of bacterial cells in the medium, *X* (mg L^–1^), is proportional to the change in lactate and sulphate concentrations, with consideration of the biomass yield terms (assuming the energy requirements for cell maintenance are negligible relative to the growth requirements).

(8) $$\frac{d\,X}{d\,t}=-Y_{L}\,\frac{d\,L}{d\,t}-Y_{S}\,\frac{d\,S}{d\,t}$$

The system consisting of Eq. 2–8 fully describes the metabolism of *D. vulgaris* under our set of assumptions. A summary of model notation is given in Table 5.

### Data Capture

Time-course data was captured from the literature using image capturing and graphical input software in MATLAB (The MathWorks^1^). The mathematical model of Noguera et al. (1998) was reconstructed using the information in the original publication. This information was near complete, the only exception being the absence of initial conditions for some of the model variables. We have therefore made some assumptions based on other information given in the paper, which has allowed us to reproduce good representations of the published model fits.

### Model Fitting

In order to determine the values of several of the parameters used in the model, model fitting to existing experimental data was performed. Time-course data from Noguera et al. (1998) was collected and used to calibrate the model and estimate parameter values.

The parameter values in Table 1 were generated by minimising the normalised sum of squared errors between the model prediction and the data. The optimisation was performed using the fminsearch routine in MATLAB (The MathWorks; see text footnote 1).

**TABLE 1:** Model parameter values.

**Parameter**		**Notation**	**Value**	**Source**	Existing estimates^*^
			**(Best fit value with 95%**		
			**confidence interval)**		
Maximum growth rates	Lactate oxidation	μ_*max,L*_	0.116 h^–1^ (0.088–1.155)	Model fitting	*t*_*d*_= 3.7 h (≈0.21 h^–1^) (Pankhania et al., 1986)
	Sulphate reduction	μ_*max,S*_	0.03 h^–1^ (0.023–0.212)	Model fitting	0.057 h^–1^ (Robinson and Tiedje, 1984) 0.15 h^–1^ (strain Marburg) (Badziong and Thauer, 1978) 0.15 h^–1^ (Reis et al., 1992)
Monod constants	Lactate	*K*_*L*_	4.5 mM (7.3–136.8)	Model fitting	1.4 mM (Pankhania et al., 1988) 29 mM (Noguera et al., 1998)
	Sulphate	*K*_*S*_	0.05 mM (0.02–0.268)	Model fitting	0.032 mM (Ingvorsen and Jørgensen, 1984) 0.21 mM (Noguera et al., 1998)
	Hydrogen	*K*_*H*_	1.69 × 10^–5^ mM (2.5 × 10^–4^–3.96 × 10^–3^)	Model fitting	0.001 mM (Kristjansson et al., 1982) 0.0019 mM (Robinson and Tiedje, 1984) 0.0014 mM (Noguera et al., 1998)
Yield parameters	Lactate	*Y*_*L*_	5.65 mg L^–1^ mM^–1^ (0.99–9.57)	Model fitting	5.3 mg L^–1^ mM^–1^ (Noguera et al., 1998) 5 mg L^–1^ mM^–1^ (Walker et al., 2009)
	Sulphate	*Y*_*S*_	4.45 mg L^–1^ mM^–1^ (2.2–19.35)	Model fitting	2.8 mg L^–1^ mM^–1^ (Noguera et al., 1998) 8.3 g mol^–1^ (strain Marburg) (Badziong and Thauer, 1978) 14.3 g cell mol^–1^ (Reis et al., 1992)
Mass transfer parameter		*k*_*L*_*a*	0.302 h^–1^ (0.182–0.914)	Model fitting	0.29 h^–1^ (Noguera et al., 1998)
Inhibitory hydrogen concentration		*H*_*max*_	0.0216 mM (0.0341–0.0821)	Model fitting	0.001 atm (≈0.0007 mM) (Junicke et al., 2015)
Stoichiometric constants	Moles of hydrogen (H_2_) produced per mole lactate oxidised	*b*_*LH*_	2.5	Assumed stoichiometries	2.5 (Thauer et al., 1977; Noguera et al., 1998) 3.5 (Keller and Wall, 2011)
	Moles of hydrogen (H_2_) consumed per mole H_2_S produced	*b*_*HP*_	5	Assumed stoichiometries	5 (Thauer et al., 1977; Noguera et al., 1998) 4.25 (Keller and Wall, 2011)
	Moles of acetate produced per mole lactate oxidised	*b*_*LA*_	1	Assumed stoichiometries	1 (Thauer et al., 1977; Noguera et al., 1998; Keller and Wall, 2011)
	Moles of H_2_S produced per mole sulphate reduced	*b*_*SP*_	1	Assumed stoichiometries	1 (Thauer et al., 1977; Noguera et al., 1998; Keller and Wall, 2011)
Henry constant		ρ_*H*_	1.364	Obtained from literature (Sander, 2015)	

^*^These estimates are obtained from different models and therefore a direct comparison cannot be made with the parameters estimated in the paper. They are listed here for reference.

### Statistical Analysis

All statistics were calculated in MATLAB using the captured data and corresponding model prediction. A Markov Chain Monte Carlo (MCMC) technique was implemented over 200,000 MCMC iterations. A non-parametric distribution was then fitted to the MCMC sample for each of the nine parameters estimated. The cumulative density function of this distribution was used to obtain a 95% confidence interval.

To compare the proposed model with the existing model of Noguera et al. (1998), we used the corrected Akaike Information Criterion (AICc) (Akaike, 1974; Hurvich and Tsai, 1989):

$$\mathrm{AICc}=2\,K-2\,\left(\log\left(ℒ\,\left(\theta \right)\right)\right)+\frac{2\,K\,\left(K+1\right)}{n-K-1}$$

where *n* is the number of data points (63), *K* is the number of parameters of the model and log⁡(ℒ(θ)) is the log likelihood function for the model. Following Burnham and Anderson (2002), we make the substitution

$$\log\left(ℒ\left(\theta \right)\right)=-\frac{1}{2}n\log{\left(\frac{\mathrm{RSS}}{n}\right)}^{2}$$

where RSS is the normalised residual sum of squares of the model fit to the data. Normalisation, i.e., division by the sample mean in the calculation of the RSS for each data set, was included to ensure the RSS value was not biassed by the scale on which each variable was measured. Finally, we also calculate the Akaike weight, *w*_*i*_, for each model as follows (Burnham and Anderson, 2002):

$$w_{i}=\frac{l_{i}}{l_{1}+l_{2}}$$

where $l_{i}=\exp\left(-\frac{1}{2}\,\left({\mathrm{AICc}}_{i}-{\mathrm{AICc}}_{m\,i\,n}\right)\right)$. Here, *i* is the model index (1 for the existing model of Noguera et al. (1998), 2 for the model presented here) and *AICc*_*min*_ represents the minimum *AICc* value of the two models.

## Results

### Model Calibration

**FIGURE 2:**
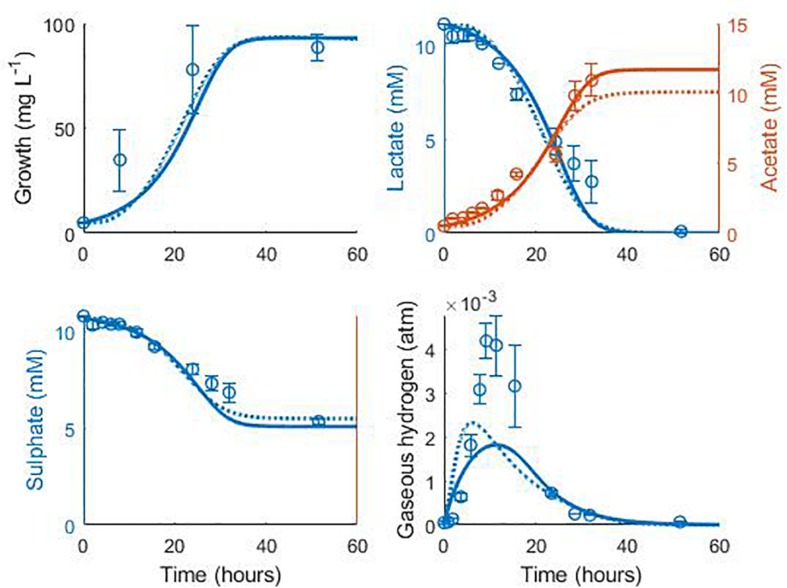
Model fits to data from Noguera et al. (1998): continuous lines display the fit of the current model; dotted lines display the fit of the model described in Noguera et al. (1998). Analysis of model fit is presented in Table 2.

**FIGURE 3:**
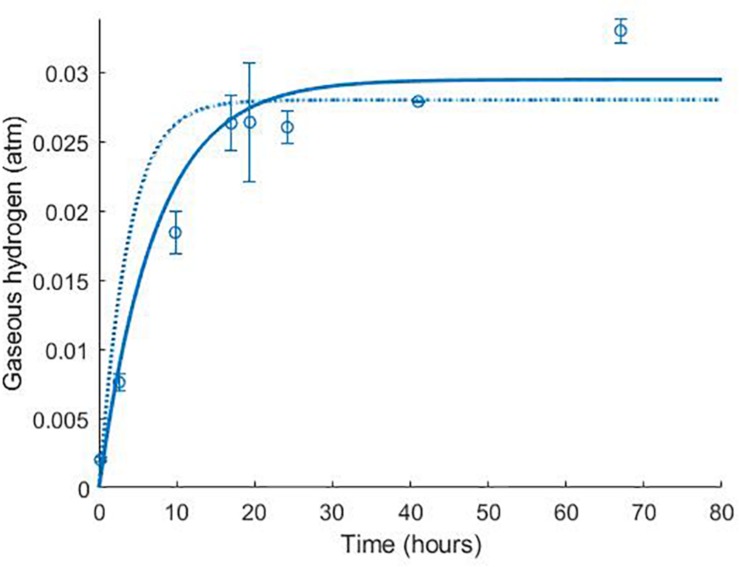
Dynamics of gaseous hydrogen in medium supplemented with 17.3 mM lactate in the absence of sulphate (Noguera et al., 1998): continuous lines display the fit of the current model; dotted lines display the fit of the model described in Noguera et al. (1998). Analysis of model fit is presented in Table 2.

Data from two separate experiments were used simultaneously to obtain parameter values for the model (Noguera et al., 1998). The first experiment involved the growth of *D. vulgaris* in medium supplemented with lactate and sulphate (Figure 2), while the second experiment took place in the absence of sulphate (Figure 3). Our mathematical model was able to describe the trends in growth and metabolite flux dynamics for both these experiments, giving comparable goodness of fit to the more complex model of Noguera et al. (1998; Table 2). The parameter values used are shown in Table 1.

**TABLE 2:** Analysis of model fits to the calibration data.

**Variable**	**Noguera et al. (1998) model**	**Current model**			
	***R*^2^**	***R*^2^**	**Pearson’s correlation coefficient (95% confidence interval)**	**CCC^†^**	**Mean bias**
**Figure 2**					
Cell concentration	0.83	0.80	0.93 (−0.28, 0.99)	0.84	−10.58 mg L^–1^
Lactate	0.95	0.96	0.98 (0.95–0.99)	0.96	−0.06 mM
Acetate	0.92	0.97	0.99 (0.94–0.99)	0.97	−0.23 mM
Sulphate	0.94	0.93	0.99 (0.94–0.99)	0.95	−0.26 mM
Gaseous hydrogen	<0	<0	0.93 (0.77–0.98)	0.57	−4.95 × 10^–4^ atm
**Figure 3**					
Gaseous hydrogen	0.83	0.96	0.98 (0.89–0.99)	0.96	−4.75 × 10^–4^ atm

*^†^CCC: Concordance correlation coefficient (Lin, 1989).*

The model of Noguera et al. (1998) uses seven model fitted parameters and a total of 20 parameters either fitted or estimated from previous experimentation, whereas our model uses nine fitted parameters and one estimated from previous experimentation, giving a total of 10. This discrepancy is due to the increased complexity of the former model, which additionally models the concentrations and gaseous partial pressures of CO_2_, H_2_S and bicarbonate, as well as the mass transfer of these molecules between the two phases, and the thermodynamics of each reaction modelled. Table 3 details the values used for the AICc calculation. The AICc value for our model was 263.2 compared to an AICc of 282.8 for the model of Noguera et al. (1998). This indicates the aptness of our model to the data considered, although more complex models may be better suited for larger and more complex data sets.

**TABLE 3:** AIC calculation values.

**Model**	***n***	***K***	**RSS**	**log⁡(ℒ(θ))**	**AICc**	**Akaike weight**
Noguera et al. (1998)	63	20	9.6095	−111.4	282.8	0.0001
This model	63	10	8.9861	−119.5	263.2	0.9999

Some of the parameters shown in Table 1 were fixed to values taken from the literature. The stoichiometric constants were fixed to correspond with the assumed stoichiometries of the reactions considered and the Henry constant for hydrogen was also obtained from the literature.

It is notable that the best fit parameter values for *K*_*L*_, *K*_*H*_ and *H*_*max*_ lie outside their respective MCMC generated 95% confidence interval. This is likely due to the difficulties in estimating half-saturation constants and maximum growth rates simultaneously, as we observed high correlation between these values. This has been observed in Monod model fitting elsewhere [for example, Muñoz-Tamayo et al. (2016)]. We therefore performed a second MCMC run in which the half-saturation constants were fixed at values obtained from the experimental literature. A comparison of the newly generated confidence intervals for the remaining fitted parameters with the original values is shown in Table 4, but the intervals are similar. We therefore analysed the sensitivity of the model prediction to variations in each parameter value (Supplementary Table S1). The model prediction for growth in medium with no sulphate, shown in Figure 3, was not notably sensitive to small changes in any fitted parameter value except for *H*_*max*_, which determines the final partial pressure of gaseous hydrogen. Contrastingly, the model fit to gaseous hydrogen shown in Figure 2 showed sensitivity to a number of parameters. Small variations in the maximum growth rates, half-saturation constant for lactate, yield values and the stoichiometric constants *b*_*LH*_ and *b*_*HP*_, all resulted in relatively large changes in the quality of fit of the model to the gaseous hydrogen data. The change in the goodness of fit to the other data types was minimal. We also found that the model fit was only slightly sensitive to small changes in the initial conditions for lactate, sulphate and bacterial concentration and insensitive to such changes in the initial conditions for other metabolites. This was in contrast to the model of Noguera et al. (1998), which we found to be disproportionately sensitive to small changes in the initial conditions for dissolved hydrogen and carbonates: variables less likely to have a strong effect on culture dynamics than lactate, sulphate and bacterial concentrations.

**TABLE 4:** Confidence interval comparisons.

**Parameter**		**Notation**	**MCMC generated 95% confidence interval**	
			**Fitted half-saturation parameters**	**Fixed half-saturation parameters**
Maximum growth rates	Lactate oxidation	μ_*max,L*_	0.088–1.155	0.02–0.145
	Sulphate reduction	μ_*max,S*_	0.023–0.212	0.021–0.171
Monod constants	Lactate	*K*_*L*_	7.3–136.8	–
	Sulphate	*K*_*S*_	0.02–0.268	–
	Hydrogen	*K*_*H*_	2.5 × 10^–4^–3.96 × 10^–3^	–
Yield parameters	Lactate	*Y*_*L*_	0.99–9.57	1.18–9.4
	Sulphate	*Y*_*S*_	2.2–19.35	1.99–17.2
Mass transfer parameter		*k*_*L*_*a*	0.182–0.914	0.313–3.724
Inhibitory hydrogen concentration		*H*_*max*_	0.0341–0.0821	0.0335–0.0797

**TABLE 5:** Model notation.

**Notation**	**Description**	**Unit**
μ_*max,L*_	Maximum growth rate for lactate	h^–1^
μ_*max,S*_	Maximum growth rate for sulphate	h^–1^
*K*_*L*_	Half-saturation constant for lactate	mM
*K*_*S*_	Half-saturation constant for sulphate	mM
*K*_*H*_	Half-saturation constant for hydrogen	mM
*Y*_*L*_	Yield term for lactate oxidation	mg L^–1^ mM^–1^
*Y*_*S*_	Yield term for sulphate reduction	mg L^–1^ mM^–1^
*H*_*max*_	Inhibitory aqueous hydrogen concentration	mM
*k*_*L*_*a*	Mass transfer coefficient	h^–1^
*b*_*LH*_	Moles of hydrogen produced per mole lactate oxidised	–
*b*_*HP*_	Moles of hydrogen utilised per mole H_2_S produced	–
*b*_*LA*_	Moles of acetate produced per mole lactate oxidised	–
*b*_*SP*_	Moles of H_2_S produced per mole sulphate reduced	–
*L*	Lactate concentration	mM
*S*	Sulphate concentration	mM
*H*_*aq*_	Aqueous hydrogen concentration	mM
*H*_*g*_	Gaseous hydrogen concentration	atm
*A*	Acetate concentration	mM
*P*	H_2_S concentration	mM
*X*	Bacterial cell concentration	mg L^–1^
*t*	Time	h
ρ_*H*_	Henry conversion constant for hydrogen	atm mM^–1^
*V*_*aq*_	Volume of the aqueous phase [50 mL for the experiments of Noguera et al. (1998), 250 mL for the experiments of da Silva et al. (2013)]	mL
*V*_*g*_	Volume of the gaseous phase [110 mL for the experiments of Noguera et al. (1998), 250 mL for the experiments of da Silva et al. (2013)]	mL

### Model Validation

**FIGURE 4:**
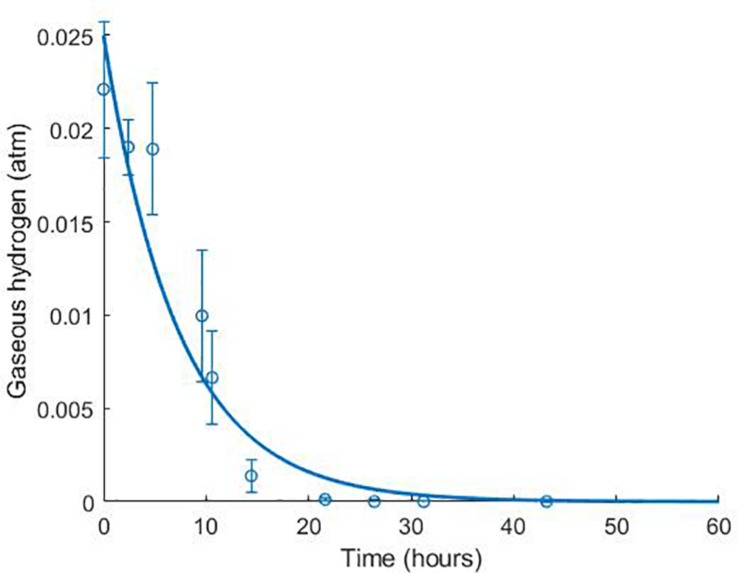
Dynamics of gaseous hydrogen in medium supplemented with 9.3 mM sulphate in the absence of lactate, with an initial hydrogen partial pressure in the gaseous phase of approximately 0.025 atm. 12 mM acetate was added as a carbon source (Noguera et al., 1998). The solid line shows the prediction of the current model. *R*^2^ = 0.91, ρ = 0.96 (0.83, 0.99), CCC = 0.92, mean bias = –0.0003 atm.

The model was validated against a number of different experimental data sources (Noguera et al., 1998; da Silva et al., 2013). Figure 4 shows the model simulation for gaseous hydrogen dynamics in medium lacking lactate, where *D. vulgaris* may only perform sulphate reduction, until the available hydrogen is depleted [data from Noguera et al. (1998)].

**FIGURE 5:**
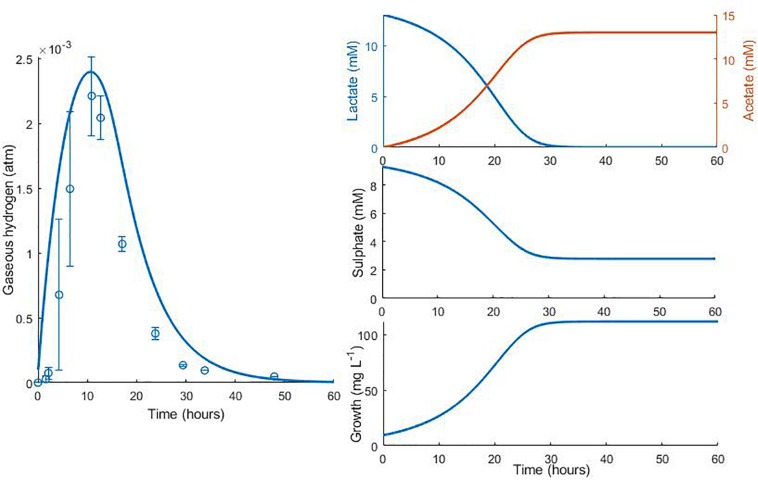
Dynamic changes in gaseous hydrogen with initial metabolite concentrations: 13 mM lactate; 9.3 mM sulphate (Noguera et al., 1998). Solid lines show the prediction of the current model. *R*^2^ = 0.70, ρ = 0.93 (0.76, 0.98), CCC = 0.77, mean bias = 0.0004 atm.

**FIGURE 6:**
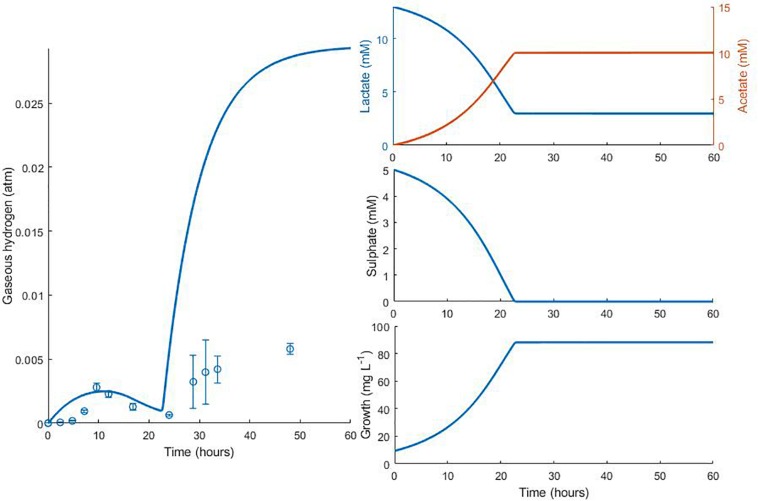
Dynamic changes in gaseous hydrogen with initial metabolite concentrations: 13 mM lactate; 5 mM sulphate (Noguera et al., 1998). Solid lines show the prediction of the current model. *R*^2^ = 0.22, ρ = 0.91 (0.72, 0.97), CCC = 0.22, mean bias = 0.0062 atm.

Figures 5, 6 show the comparison between the model prediction and experimental data from further validation experiments, with altered initial conditions [data from Noguera et al. (1998)]. Unfortunately, for these and the experiments from which Figures 3, 4 were generated, data for aqueous metabolite concentrations and bacterial growth are unavailable, so we cannot verify the model predictions for these variables. We also have no information regarding the concentration of bacteria at the beginning of the experiment, therefore 9.4 mg L^–1^, the initial bacterial concentration in previous experiments, was assumed.

The model predicts the full utilisation of lactate and only partial consumption of sulphate in Figure 5, but is not able to capture the delay in hydrogen accumulation in the headspace observed in the first few hours of the experiment. The same is true of Figure 6. Here, the model accurately predicts the final concentration of lactate remaining in the medium at the end of the experiment as 2.98 mM, compared to the observed value of 2.58 mM. However, the model overpredicts the gaseous hydrogen accumulation. Under our model assumptions we expect hydrogen to accumulate to the inhibitory level, whereas in the experiment hydrogen production was far lower. Given that the model accurately predicted the lactate degradation, this would imply that less hydrogen is produced under the conditions shown in Figure 6 than under the assumed stoichiometry. Hydrogen accumulation was not measured after 48 h in the experiment, therefore it is not possible to know whether and at what point hydrogen accumulation peaks.

**FIGURE 7:**
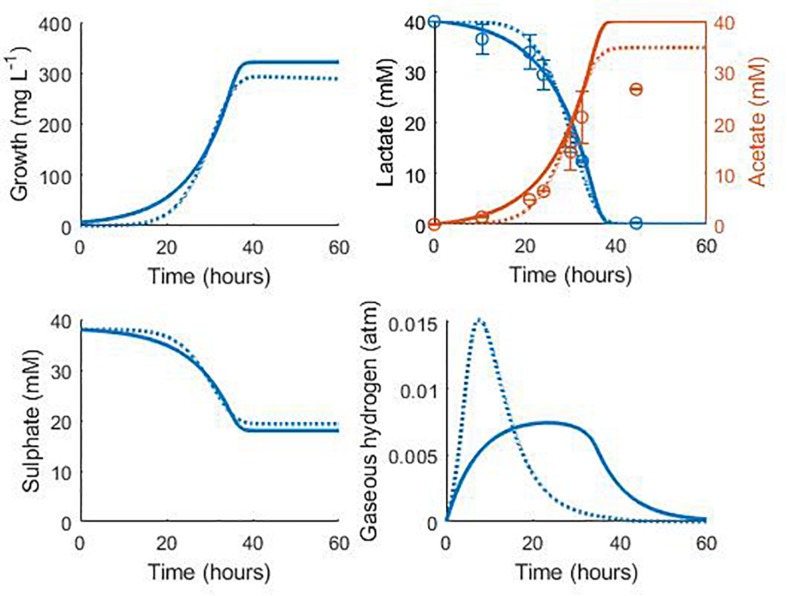
Model prediction for the consumption of lactate and production of acetate in the experimental work of da Silva et al. (2013): continuous lines display the fit of the current model; dotted lines display the fit of the model described in Noguera et al. (1998). See text for full explanation. Continuous line fit: Lactate: *R*^2^ = 0.98, ρ = 0.99 (0.95, 0.99), CCC = 0.98, mean bias = 1.2 mM; Acetate: *R*^2^ = 0.82, ρ = 0.99 (0.93, 0.99), CCC = 0.88, mean bias = 4.12 mM. Dotted line fit: Lactate: *R*^2^ = 0.98, ρ = 0.99 (0.96, 0.99), CCC = 0.98, mean bias = 1.32 mM; Acetate: *R*^2^ = 0.90, ρ = 0.99 (0.97, 0.99), CCC = 0.93, mean bias = 2.08 mM.

Figure 7 shows the validation of the both our model and that of Noguera et al. (1998) against separate experimental data for *D. vulgaris* Hildenborough, taken from da Silva et al. (2013). The experimental starting concentration of bacteria was not stated for this data set, so we fitted this value to the data with all other parameters fixed at their previously determined values. This gave an initial bacterial concentration of 6.75 mg L^–1^ for our model and 0.038 mg L^–1^ for the model of Noguera et al. (1998). As shown in Figure 7, the models performed similarly with their respective initial bacterial concentrations, with the exception of the gaseous hydrogen prediction, and both accurately captured the rate of lactate degradation and acetate production with no alteration to the parameter values obtained during model calibration. The large discrepancy between the obtained initial bacterial concentrations for the two models prompted further investigation. The initial optical density (OD) recorded for this experiment was approximately 0.025 (da Silva et al., 2013). No calibration to other units was performed by these authors and few exist in the literature for *Desulfovibrio* strains, but Bernardez and de Andrade Lima (2015) suggested a conversion of: dry weight (mg) = exp (5.12 OD–4.987), which gives an approximate initial bacterial concentration for this experiment of 7.76 mg L^–1^. Although the conditions under which this conversion was derived differ from the experiment of da Silva et al. (2013), this estimate compares well to that of our model.

The final acetate measurement in Figure 7 was not predicted by either model, and it is not clear to where the remaining carbon from lactate degradation was directed in this experiment. *D. vulgaris* has the potential to use acetyl-CoA, an intermediate on the lactate oxidation pathway, in the biosynthesis of certain branched-chain amino acids and fatty acids, as well as in an incomplete citric acid cycle (Heidelberg et al., 2004), but only the metabolites shown in Figure 7 were measured. However, separate experiments by these authors with concentrated cell suspensions found the expected 1:1 ratio of lactate degraded to acetate produced (da Silva et al., 2013).

## Discussion

This model provides a simpler mathematical representation of SRB metabolism than is currently available in the literature (Noguera et al., 1998), with similar predictive capability. As such, it can be more easily adapted to specific strains and culture conditions, not limited to SRB of the human GIT. The inclusion of further characteristics of specific SRB strains could be realised with the addition of further terms to existing equations, or the inclusion of further equations if additional metabolites were considered. For example, complete growth inhibition of a SRB strain due to sulphide concentrations above 16.1 mM has been shown previously (Reis et al., 1992). Acetate inhibition has also been investigated for SRB, with approximately 54 mg L^–1^ undissociated acetic acid (≈45.9 mM acetate) resulting in 50% growth inhibition (Reis et al., 1990). Both these concentrations are greater than those measured in the experiments used here, and the H_2_S concentration is greater than that reported in faeces (Magee et al., 2000). However, the model could be adjusted to include inhibition terms for acetate and H_2_S for application of the model to more extreme environments. These terms could take the form used here for hydrogen inhibition, but alternative inhibition terms could be more appropriate and should be assessed by model fitting (see Han and Levenspiel (1988) for a list of inhibition terms and a generalised form). At present, we are not aware of any time-course data involving such concentrations of these metabolites with which to parameterise the model.

It would also be useful to investigate experimentally the influence of bicarbonate on the growth rate of SRB. Several human-associated bacterial strains have shown reduced growth rates when exposed to 100 mM of bicarbonate in monoculture (Dobay et al., 2018). This molecule was also shown to disrupt biofilm formation in selected strains. *D. vulgaris* is a biofilm forming organism (Clark et al., 2007), but no SRB were studied in the bicarbonate inhibition experiments, so we cannot make any inference about the influence of this molecule on growth rates in our model. However, following the expected stoichiometry of the *D. vulgaris* metabolic pathways, we would anticipate less than 20 mM of bicarbonate could be produced in the experiments of Noguera et al. (1998), and up to 40 mM in the experiments of da Silva et al. (2013), considerably lower than those found to be growth limiting in Dobay et al. (2018). Bicarbonate is secreted into the gastrointestinal lumen in humans, reaching comparable concentrations to those expected in these experiments: bicarbonate concentration at the start of the colon is estimated at around 30 mM (Gennari and Weise, 2008). Further experimental investigation is needed to determine whether, and to what extent, bicarbonate may be growth limiting to SRB before it can be included in a model.

Time-course data is also unavailable for the use of acetate as a carbon source by SRB, which has been shown in the absence of lactate [for example, Pankhania et al. (1986)]. We expect that acetate uptake is occurring in the data shown in Figure 4, as it is the sole available carbon source in the medium, but this was not measured. Experiments measuring acetate concentrations over time when this is the sole carbon source are required to determine the parameter values of acetate utilisation via model fitting.

Modelling mass transfer in experiments such as those described here is challenging. Due to limited available experimental data, we chose to use a simple mass transfer model to minimise the number of fitting parameters required. Mass transfer is modelled under the assumption of linear dynamics, but without knowledge of the concentration of dissolved hydrogen it is unclear how much this assumption biases the model. The model may be more limited in its ability to accurately capture hydrogen transfer between phases than other, more complex model structures (Kadic and Heindel, 2014). This simple structure may be partially responsible for the sensitivity of the gaseous hydrogen model fit to small changes in some of the parameter values of the model. However, we believe that the model fit to the lactate, acetate and sulphate data are of greater importance than those of gaseous hydrogen and bacterial growth for several reasons. The apparent initial lag phase in the gaseous hydrogen data from the experiments considered here was not captured by our model, despite the good fit to the data for other metabolites. While the inclusion of a lag phase in the model could rectify this aspect, such an addition would complicate a model that we wish to keep parsimonious and we also do not have a probable physiological cause for such a lag. The experimental data shows large variation in gaseous hydrogen pressure between replicates in both the calibration and validation datasets. The data for the concentration of bacterial cells in the medium is similarly limited. Only two measurements were taken during the exponential growth phase in Figure 2, and the error on both of these measurements is greater than 25% of the mean value. It is also unclear how reliable the initial value for cell concentration is, since this was assumed from the inoculum rather than measured. Although our model proved only slightly sensitive to certain initial condition values, measuring the initial concentrations of both cells and metabolites would be of great value. The data for lactate, acetate and sulphate concentrations are more complete and more repeatable, encouraging emphasis on the model fit to these data.

Uncertainty remains in the field around the nature of hydrogen production and use by SRB. Previously, there have been arguments both for and against its status as a mandatory intermediate in the simultaneous oxidation of organic compounds and reduction of sulphate, as well as the role of various hydrogenase enzymes (Keller and Wall, 2011; Rabus et al., 2013). The importance of hydrogen in the reduction of sulphate has also been shown differ between SRB species [see review by Rabus et al. (2015)]. We believe that one of the strengths of the model is its avoidance of any biassing assumption about the nature of these relationships by using our two hydrogen compartments, aqueous and gaseous, as a method to represent hydrogen equivalents that are immediately available for use in sulphate reduction or not, respectively.

The mathematical model presented here is simpler in its construction than previous attempts to capture SRB dynamics. Our model uses nine fitted parameters (10 parameters in total), compared to seven fitted and three experimentally estimated parameters (20 parameters in total) in Noguera et al. (1998), and seven differential equations compared with ten in Noguera et al. (1998). Our model also shows good fits to experimental data as assessed by common measures for model analysis for two *D. vulgaris* strains from several independent experiments under varied conditions. While the model of Noguera et al. (1998) considers more factors, including the thermodynamics of the conversions performed by the bacteria and the concentrations of a greater number of metabolites, these inclusions can be limiting when investigating the metabolism of SRB in environments where knowledge of these factors is not available. For example, application of the model of Noguera et al. (1998) to the human GIT would be challenged by host influences on variables. The applied model would need to consider appropriate representation of bicarbonate and CO_2_ when including secretion and absorption by the host, as well as the implications of host metabolite absorption on the modelled thermodynamic inhibition of the metabolic reactions. By contrast, the relative simplicity of our model means it can more easily be adapted to the specific environmental conditions of the GIT and has greater flexibility for the inclusion of additional influences upon the metabolism of these bacteria. In this way the model could be adapted to provide a representative model for the SRB functional group in the GIT.

Regarding dynamics in the GIT, current existing data from rodent models support the increased efficiency of carbohydrate breakdown by saccharolytic bacteria in the presence of either a methanogen or acetogen due to hydrogen metabolism by these microbes (Samuel and Gordon, 2006; Rey et al., 2010). However, there is no such evidence for the SRB, although in theory the same role could be filled by these bacteria (Smith et al., 2018). This may be due to competition for other substrates, which could be investigated using the model presented here in combination with existing models for saccharolytic bacteria [such as Kettle et al. (2015)].

It is our intention to use the SRB model presented here as part of a larger model including other hydrogenotrophic and hydrogenogenic microbes of the human GIT, to examine the role of hydrogen in this environment. Mathematical models for the GIT microbiota are available (Muñoz-Tamayo et al., 2010; Kettle et al., 2015, 2017), but as yet do not consider the action of SRB. The inclusion of this functional group may further enhance their predictive capabilities and could eventually be used to address the role of the SRB in human nutrition and health. Such community modelling should not be limited to the GIT, as the combination of models such as that presented here with similar structures for methanogens and reductive acetogens may reveal information about the cross-feeding and competitive relationships between these hydrogenotrophs in other environments.

## Data Availability

The data supporting the conclusions of this manuscript will be made available by the authors, without undue reservation, to any qualified researcher.

## Author Contributions

NS performed the mathematical modelling and drafted the manuscript. All authors contributed to model and manuscript revision, and read and approved the submitted version.

## Conflict of Interest Statement

The authors declare that the research was conducted in the absence of any commercial or financial relationships that could be construed as a potential conflict of interest.

## Footnotes

1 www.mathworks.com
