# Supplementary material for: A Mathematical Model for the Hydrogenotrophic Metabolism of Sulphate-Reducing Bacteria
Source: Front Microbiol. 2019 Jul 17;10:1652. doi: 10.3389/fmicb.2019.01652 (PMC6653664; doi:10.3389/fmicb.2019.01652)
Supplement: Supplementary file 1 [file Table_1.DOCX]

Supplementary table S1. Percentage change in mean bias value in response to a one-at-a-time 10% variation in initial condition or parameter value to assess model sensitivity. + indicates increase in mean bias, - indicates a decrease.

|  | **Figure 2** | | | | | **Figure 3** |
| --- | --- | --- | --- | --- | --- | --- |
|  | Cell concentration | Lactate | Acetate | Sulphate | Gaseous hydrogen | Gaseous hydrogen |
| **Initial condition variation** |  |  |  |  |  |  |
| Lactate +10% | +13% | +111% | +25% | +38% | -13% | 0% |
| Lactate -10% | +5% | +45% | +29% | -27% | +24% | 0% |
|  |  |  |  |  |  |  |
| Sulphate +10% | 0% | 0% | 0% | 140% | 0% | -1% * |
| Sulphate -10% | 0% | 0% | 0% | 271% | 0% | - |
|  |  |  |  |  |  |  |
| Aqueous hydrogen +10% | 0% | 0% | 0% | 0% | 0% | 0% |
| Aqueous hydrogen -10% | 0% | 0% | 0% | 0% | 0% | 0% |
|  |  |  |  |  |  |  |
| Gaseous hydrogen +10% | 0% | 0% | 0% | 0% | 0% | 0% |
| Gaseous hydrogen -10% | 0% | 0% | 0% | 0% | 0% | 0% |
|  |  |  |  |  |  |  |
| Acetate +10% | 0% | 0% | -4% | 0% | 0% | 0% * |
| Acetate -10% | 0% | 0% | 7% | 0% | 0% | - |
|  |  |  |  |  |  |  |
| H_2_S +0.1 mM * | 0% | 0% | 0% | 0% | 0% | 0% |
|  |  |  |  |  |  |  |
| Cell concentration +10% | -9% | 8% | -2% | 13% | 1% | - |
| Cell concentration -10% | 11% | -3% | 16% | -13% | 0% | - |
|  |  |  |  |  |  |  |
| **Parameter variation** |  |  |  |  |  |  |
| $\mu_{max,L}$ +10% | -6% | 17% | 9% | 15% | -16% | 1% |
| $\mu_{max,L}$ -10% | 13% | -14% | 26% | -30% | 65% | -1% |
|  |  |  |  |  |  |  |
| $\mu_{max,S}$ +10% | -10% | 8% | -2% | 14% | 63% | 0% |
| $\mu_{max,S}$ -10% | 18% | -4% | 29% | -29% | 4% | 0% |
|  |  |  |  |  |  |  |
| $K_{L}$ +10% | 3% | -11% | 7% | -10% | 20% | 0% |
| $K_{L}$ -10% | -3% | 10% | 1% | 9% | -15% | 0% |
|  |  |  |  |  |  |  |
| $K_{S}$ +10% | 0% | 0% | 0% | 0% | 0% | 0% |
| $K_{S}$ -10% | 0% | 0% | 0% | 0% | 0% | 0% |
|  |  |  |  |  |  |  |
| $K_{H}$ +10% | 0% | 0% | 0% | 0% | 0% | 0% |
| $K_{H}$ -10% | 0% | 0% | 0% | 0% | 0% | 0% |
|  |  |  |  |  |  |  |
| $Y_{L}$ +10% | 7% | -7% | 4% | -6% | 62% | 1% |
| $Y_{L}$ -10% | 7% | -4% | 3% | -5% | -5% | 0% |
|  |  |  |  |  |  |  |
| $Y_{S}$ +10% | 15% | -1% | 18% | -17% | -7% | 0% |
| $Y_{S}$ -10% | -8% | 1% | -7% | 7% | 66% | 0% |
|  |  |  |  |  |  |  |
| $k_{L}a$ +10% | 0% | 0% | 0% | 0% | 0% | 22% |
| $k_{L}a$ -10% | 0% | 0% | 0% | 0% | 3% | -18% |
|  |  |  |  |  |  |  |
| $H_{max}$ +10% | 0% | 0% | 0% | 0% | -5% | 78% |
| $H_{max}$ -10% | 0% | 0% | 0% | 0% | 4% | 11% |
|  |  |  |  |  |  |  |
| $b_{LH}$ +10% | 15% | -1% | 18% | 23% | -4% | 0% |
| $b_{LH}$ -10% | -8% | 1% | -7% | -24% | 66% | -1% |
|  |  |  |  |  |  |  |
| $b_{HP}$ +10% | -7% | 2% | -7% | -22% | 63% | 0% |
| $b_{HP}$ -10% | 17% | -1% | 20% | 24% | 2% | 0% |
|  |  |  |  |  |  |  |
| $b_{LA}$ +10% | 0% | 0% | 26% | 0% | 0% | 0% |
| $b_{LA}$ -10% | 0% | 0% | 36% | 0% | 0% | 0% |
|  |  |  |  |  |  |  |
| $b_{SP}$ +10% | 0% | 0% | 0% | 0% | 0% | 0% |
| $b_{SP}$ -10% | 0% | 0% | 0% | 0% | 0% | 0% |
|  |  |  |  |  |  |  |
| $\rho_{H}$ +10% | 0% | 0% | 0% | 0% | -5% | 79% |
| $\rho_{H}$ -10% | 0% | 0% | 0% | 0% | 5% | 11% |

*H_2_S concentration was increased by 0.1 mM rather than a 10% variation since its initial concentration was assumed to be 0 mM for the model fitting in Figure 2. The same was performed for sulphate and acetate concentrations for the sensitivity analysis of the model fit in Figure 3.
